# *Dugesia sicula* (Platyhelminthes, Tricladida): the colonizing success of an asexual Planarian

**DOI:** 10.1186/1471-2148-13-268

**Published:** 2013-12-11

**Authors:** Eva Mª Lázaro, Marta Riutort

**Affiliations:** 1 Departament de Genètica, Facultat de Biologia and Institut de Recerca de la Biodiversitat (IRBio), Universitat de Barcelona, Barcelona, Catalonia, Spain

**Keywords:** Heteroplasmy, Fissiparity, Asexual, Recent colonization, Mediterranean basin, Trade routes, Niche modelling

## Abstract

**Background:**

*Dugesia sicula* is the only species of its genus not presenting an endemic or restricted distribution within the Mediterranean area. It mostly comprises fissiparous populations (asexual reproduction by body division and regeneration), most likely sexually sterile, and characterized by an extremely low genetic diversity interpreted as the consequence of a recent anthropic expansion. However, its fissiparous reproduction can result in an apparent lack of diversity within the species, since genetic variation within individuals can be as large as between them because most individuals within a population are clones. We have estimated haplotype and nucleotide diversity of *cytochrome oxidase I* within and among individuals along the species distribution of a broad sample of *D. sicula*, including asexual and the two only sexual populations known today; and predicted its potential distribution based on climatic variables. Our aim was to determine the centre of colonisation origin, whether the populations are recent, and whether the species is expanding.

**Results:**

The species presents 3 most frequent haplotypes, differing in a maximum of 11 base pairs. As expected from their fissiparous mode of reproduction, in half of all the analysed localities many individuals have multiple heteroplasmic haplotypes. The distribution of haplotypes is not geographically structured; however, the distribution of haplotypes and heteroplasmic populations shows higher diversity in the central Mediterranean region. The potential distribution predicted by climatic variables based modelling shows a preference for coastal areas and fits well with the observed data.

**Conclusions:**

The distribution and frequency of the most frequent haplotypes and the presence of heteroplasmic individuals allow us to gain an understanding of the recent history of the species, together with previous knowledge on its phylogenetic relationships and age: The species most probably originated in Africa and dispersed through the central Mediterranean. After one or multiple populations became triploid and fissiparous, the species colonized the Mediterranean basin, likely both by its own means and helped by human activities. Its present distribution practically fulfils its potential distribution as modelled with climatic variables. Its prevalence in coastal regions with higher water temperatures predicts a likely future expansion to northern and more interior areas following the increase in temperatures due to climate change.

## Background

*Dugesia sicula* Lepori, 1948 is a freshwater planarian (phylum Platyhelminthes, order Tricladida, suborder Continenticola) typically found in ponds, streams and springs close to the Mediterranean coast. The first record of *D. sicula* described a population comprising both sexual and asexual (fissiparous) individuals in Catania, Sicily [1]. Fissiparity is an asexual form of reproduction that involves transversal division of the individuals into two fragments and subsequent regeneration of the absent structures. Fissiparous planarians do not develop a copulatory apparatus, which precludes the assignment of these populations to any species since this structure provides most of the defining characters for species description in planarians. However, the morphological characters of the copulatory apparatus of the sexual specimens allowed the species to be described. *D. sicula* was subsequently found on the islands of Elba [2] and Mallorca [3]. None of the originally described sexual populations can be currently found [4]; but, some sexual populations in northern Africa (Tunisia and Algeria) have been recently reported [5-7]. Moreover, a molecular-based study [8] showed that most of the numerous fissiparous populations of genus *Dugesia* present in the western Mediterranean belong to *D. sicula*. Under certain conditions (mostly in the laboratory) triploid fissiparous planarians can develop hyperplasic ovaries and a copulatory apparatus [4,9,10], these individuals are known as ex-fissiparous. In *D. sicula*, it has been observed that some ex-fissiparous individuals laid cocoons but remained sterile [4,9-11], which indicated that asexual populations have lost the capability to sexually reproduce.

The literature suggests that *D. sicula* is widely distributed in the Mediterranean area from Morocco to Greece [4,9,12], and our own observations show that this species mostly comprise fissiparous populations, most likely sexually sterile, distributed throughout the Mediterranean, as well as the Canary Islands, being the only species of this genus in the Mediterranean with a such a wide distribution. Contrasting with this wide distribution the species presents extremely low variability for the COI gene [8] among populations located hundreds of kilometres apart. These two features (wide distribution and low genetic variability) could be a consequence of a recent expansion promoted by human activities, as has been shown for other organisms [13]. Alternatively, the low levels of variability could be exclusively a consequence of their fissiparous reproduction, as described in *Schmidtea mediterranea*[14] and other organisms [15], and its wide distribution may be a consequence of its active spread over the years. In both cases, the hypotheses contrast with the poor dispersion capability assumed for freshwater planarians [16,17]. Freshwater planarians do not exhibit larval dispersal stages or forms resistant to desiccation; these individuals thus require contiguous freshwater bodies to survive and disperse [16,17]. However, introductions due to human activities have been documented in other freshwater planarians, such as *Girardia tigrina*[18] and *Schmidtea polychroa*[19] and more recently have also been proposed for other *Dugesia* species [20], but in all these cases the introduced animals either have a restricted distribution (*Dugesia*) or its introduction and progression in the new areas has been followed by scientist.

Fissiparous reproduction can have effects on the distribution of genetic variability within the populations and even on individuals that could perhaps help disentangle this situation. Regeneration after fission is driven by neoblasts [21,22], which are pluripotent stem cells. A new mutation in a neoblast would expand in the individual when this neoblast generates new tissues after the fission of the animal, thus increasing frequency of the mutation in the individual. Following replication via several fission cycles, the new mutation could disappear or spread in the population of cells within the individual and the organism’s population by genetic drift (Additional file 1: Figure S1). In this manner, we would expect to observe cells with different sequences within one individual. This could be considered a special case of heteroplasmy that differs from cases in which heteroplasmy is transmitted sexually or parthenogenetically through the mitochondria present in the oocyte (and the spermatozoon in some cases) where all cells from a new individual will present the same haplotypes. Heteroplasmy has been detected in many species [23], and some studies in humans have shown it increases with age in somatic cells and is more frequent in certain tissues, such as muscle tissue, most likely because of increases in the number of mutations caused by the presence of oxidative radicals [23,24]. Heteroplasmy has also been detected in COI sequences of fissiparous populations of *D. japonica*[25]. Due to their characteristics, in fissiparous planarians the somatic heteroplasmy would be maintained in populations and increased over time.

If this situation holds in fissiparous populations of *D. sicula*, the analysis of their genetic variability could help in discriminating between a recent or ancient origin of the species populations and hence provide clues to the origin of its distribution. The absence or low frequency of heteroplasmic specimens in a specific locality of fissiparous reproducing individuals could be interpreted as a consequence of a recent bottleneck, potentially because of recent colonisation by a few individuals or recovery from a recent population-wide decrease. The alternate condition, the presence of many heteroplasmic individuals, particularly those exhibiting a high number of different low-frequency haplotypes, could then potentially be related to aged individuals and populations, although a recent colonisation by highly heteroplasmic individuals could also explain this outcome. Following the same reasoning, one would not expect to find heteroplasmy, or different copies of mitochondrial genes within an individual in sexual organisms.

In the present study, we analysed the genetic diversity and structure (using COI sequences) of a broad sampling of *D. sicula* from the Mediterranean, and the potential species’ distribution was modelled to determine (1) whether intrapopulation and intraindividual nucleotide and haplotype diversity varies within the distribution; (2) whether the species is currently expanding. With this data we expect to be able to answer questions on where the centre of origin of *D. sicula*’s present distribution is located, if the species has expanded recently assisted by humans and whether it is expected to continue its expansion.

## Methods

### DNA extraction and sequence amplification

**Figure 1:**
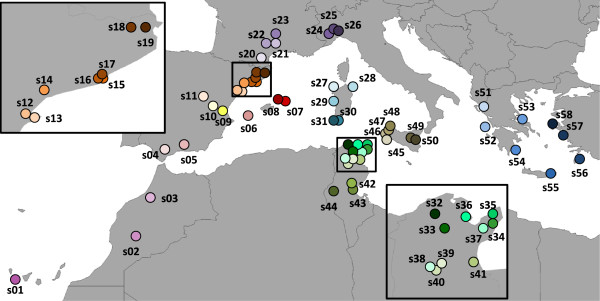
**Sampling sites for *D. sicula *populations within the Mediterranean basin.** Specimens from s39 and s36 exhibited sexual reproduction. The individuals from s55 possessed a copulatory apparatus. s43 was collected in the same pond where *D. maghrebiana* has been reported. s56 cohabited with *D. elegans*, and s25 cohabited with *D. liguriensis*. Details regarding the sampling sites and collectors are provided in Table 1.

Fifty-eight populations were sampled from the Mediterranean coast (Table 1 and Figure 1). Individuals from the Boussadia and Soukra populations (s39 and s36) in Tunisia reproduce sexually [5,6]. Individuals collected from Crete (s55), Greece, also possess a copulatory apparatus. Approximately 15 individuals (between 1 and 20, Additional file 2: Table S1) were analysed from each location. High-molecular-weight DNA was purified from live or ethanol-fixed specimens using DNAzol reagent (Molecular Research Center Inc., Cincinnati, OH). A 711-bp fragment of the mitochondrial gene COI was amplified and sequenced with specific primers (Tables 2 and 3). In some individuals, the sequence had to be obtained in two non-overlapping fragments since we were not able to obtain amplifications in a single fragment. Sequencing was performed using Big Dye (3.1, Applied Biosystems, Norwalk, CT, USA) and an automated sequencer, ABI Prism 3730 Applied Biosystems/Hitachi (Unitat de Genòmica dels Serveis Científico-Tècnics de la UB) or at Macrogen Inc. (Korea). The DNA sequences were aligned in BioEdit [26] by sight based on the amino acid sequence and submitted to GenBank [GenBank: KC536630 to KC536644].

**Table 1:** Sampling location details

**Code**	**Origin**	**Collectors and sampling date**	**Latitude**	**Longitude**
s01	PN Garajonay, La Gomera, Canary Is.	E. Mateos, 2010	28.1290	-17.2515
s02	Setti Fatma, Ourika, Morocco	A. H. Harrath, M. Yacoubi, 2009	31.2192	-7.6755
s03	Morocco	P. Aguilera, C. Hernando, I. Ribera, 2007	33.5498	-6.7486
s04	Andalusia, Spain	M. Vila-Farré, 2006	36.8070	-5.3264
s05	River Chíllar, Malaga, Spain	N. Bonada, 2008	36.7451	-3.8769
s06	Es Corralassos, Eivissa, Balearic Is.	C. Maritur, 2006	38.9243	1.4362
s07	Algendar, Menorca, Balearic Is.	S. Pons, 2006	39.8730	4.2090
s08	Binimel · là, Salairó, Menorca, Bal. Is.	S. Pons, 2006	40.0535	4.0580
s09	Antella, Valencia, Spain	M. Vila-Farré, L. Leria, E. M. Lázaro, 2012	39.0792	-0.5926
s10	Casas del Río, Valencia, Spain	M. Vila-Farré, L. Leria, E. M. Lázaro, 2012	39.2967	-1.1349
s11	Júcar, Villalba de la Sierra, Cuenca, Spain	M. Vila-Farré, L. Leria, E. M. Lázaro, 2012	40.2266	-2.0894
s12	Ullals, Tarragona, Spain	M. Vila-Farré, L. Leria, E. M. Lázaro, 2012	40.6912	0.6960
s13	Torre de la Carrova, Tarragona, Spain	M. Vila-Farré, L. Leria, E. M. Lázaro, 2012	40.7527	0.5666
s14	Duesaigües, Tarragona, Spain	M. Vila-Farré, L. Leria, E. M. Lázaro, 2012	41.1516	0.9283
s15	Tres Pins Nursery, Montjuïc, Barcelona, Spain	M. Riutort, E. M. Lázaro, 2006	41.3676	2.1620
s16	Montjuïc, Barcelona, Spain	M. Vila-Farré, 2012	41.3677	2.1622
s17	P. Laberint, Barcelona, Spain	M. Vila-Farré, 2011	41.4400	2.1451
s18*	Orfes, Girona, Spain	M. Riutort, 1996	42.1717	2.8679
s19	Fluvià, Girona, Spain	M. Riutort, 2011	42.1610	2.9585
s20	Le Pont de Reynès, Languedoc-Roussillon, France	M. Vila-Farré, L. Leria, E. Solà, 2011	42.4958	2.7148
s21	Lunaç, Languedoc-Roussillon, France	M. Vila-Farré, L. Leria, E. Solà, 2011	43.7082	3.1955
s22	Prades-le-Lez, Languedoc-Roussillon, France	M. Vila-Farré, L. Leria, E. Solà, 2011	43.6841	3.8605
s23	River Herault, France	M. Riutort, E. M. Lázaro, 2009	43.9080	3.7371
s24	Invrea, Liguria, Italy	M. Pala, 2011	44.3831	8.6096
s25^1^	Bisagno, Liguria, Italy	M. Pala, 2011	44.4477	9.0011
s26	Rapallo, Genova, Italy	M. Pala, 2011	44.3522	9.2308
s27*	Isola de l’Asinara, Sardinia, Italy	M. Pala, 2005	41.0707	8.3198
s28*	Isola de Tavolara, Sardinia, Italy	M. Pala, 2005	40.9093	9.6093
s29*	Torre Argentina, Sardinia, Italy	M. Pala, 2005	40.3282	8.4610
s30*	Acqua sa Murta, Sardinia, Italy	M. Pala, 2005	39.0768	8.4238
s31*	Acqua sa Canna, Sardinia, Italy	M. Pala, 2005	39.0638	8.4543
s32	River Joumine, Tunisia	R. Sluys, A. H. Harrath, 2006	36.9871	9.6038
s33*	Fourna, Tunisia	M. Charni, 2007	36.6935	9.8306
s34*	Lebna, Tunisia	M. Charni, 2007	36.7385	10.9220
s35*	Kherba, Tunisia	M. Charni, 2007	36.8822	10.9041
s36^2^	Soukra, Tunisia	M. Charni, 2007	36.8685	10.2656
s37*	Chiba, Tunisia	M. Charni, 2007	36.6970	10.7718
s38*	Siliana, Tunisia	M. Charni, 2007	36.0822	9.3878
s39^2^	Boussadia, Tunisia	M. Charni, 2007	36.1011	9.6118
s40*	Lakhmes, Tunisia	M. Charni, 2007	35.9978	9.4813
s41	Aïn Ghanim, Tunisia	A. H. Harrath	36.0589	10.4025
s42	Leben, Tunisia	A. H. Harrath, 2007	34.5801	9.8849
s43^3^	Gabès, Tunisia	R. Sluys, A. H. Harrath, 2006	34.1739	10.0133
s44	Aïn Chebika, Tunisia	M. Riutort, 2008	33.9283	8.1235
s45	Fra. Di Marsala, O Sossio, Sicily	M. Pala, G. A. Stocchino, E. M. Lázaro, 2009	37.7786	12.4595
s46	Marausa, Sicily	M. Pala, G. A. Stocchino, E. M.Lázaro, 2009	37.9869	12.5345
s47*	Sorgente, Sicily	M. Pala, G. A. Stocchino, E. M. Lázaro, 2009	37.9866	12.9005
s48	Castellammare del Golfo, Sicily	M. Pala, G. A. Stocchino, E. M. Lázaro, 2009	38.0213	12.9069
s49	Ponte Saraceni on river Simeto, Sicily	M. Pala, 2006	37.7012	14.8001
s50	Casale del Simeto, Sicily	M. Pala, 2007	37.5804	14.8737
s51	Smertos-Filiates, Greece	E. Solà, K. Gritzalis, 2010	39.6196	20.2537
s52	Cephalonia, Greece	R. Sluys, 2009	38.1591	20.7263
s53	Kirinthos-Mandoudi, Euboea, Greece	E. Solà, 2010	38.8024	23.4473
s54	Nomia-Kalives, Peloponnese, Greece	E. Solà, J. Solà, 2010	36.6555	22.9956
s55^4^	Crete, Greece	E. Solà, E. Mateos, 2009	35.0803	25.6508
s56^5^	Rhodes, Greece	E. Solà, E. Mateos, 2009	36.3371	28.0625
s57	Samos, Greece	M. Vila-Farré, 2010	37.7767	26.7258
s58^6^	Chios, Greece	M. Vila-Farré, 2010	38.5273	25.8705

All populations were identified as *D. sicula* by their COI sequence except populations marked with * that were previously identified by karyotype; ^1^ Cohabited with *D. liguriensis*; ^2^ Sexual populations with karyotype 2n = 18; ^3^ Collected in the same pond where *D. maghrebiana* has been reported; ^4^ Individuals with copulatory apparatus; ^5^ Cohabited with *D. elegans*; ^6^ Exfissiparous.

**Table 2:** Primers used for amplification and sequencing

**Name**	**Sequence 5′-3′**	**An. Temp. (°C)**^**1**^	**Source**
BarT (F)*	ATGACDGCSCATGGTTTAATAATGAT	43	Álvarez-Presas *et al.*, [27]
COIF (F)	CCNGGDTTTGGDATDRTWTCWCA	45	Lázaro *et al.*[8]
COIR (R)	CCWGTYARMCCHCCWAYAGTAAA	45	Lázaro *et al.*[8]
COIbarc_plat_R (R)	TAATTAAAATATAAACCTCAGGATG	40	Lázaro *et al.*[8]
BARSI (F)**	GATAGGCGGTTTTGGTAAATGG	50	This study

F forward, R reverse, *Only for amplification, **Only for sequencing ^1^ Annealing Temperature.

**Table 3:** Length of amplified fragments

**Primers**	**Sequence length**
BarT*/BARSI** + COIR	711
BarT*/BARSI** + COIbarc_plat_R	396
COIF + COIR	228

*Only for amplification, **Only for sequencing.

### Intraindividual polymorphism

To analyse the presence of multiple copies of the mitochondrial genome within individuals, some of the PCR products were cloned using an HTP TOPO TA Cloning Kit for sequencing (Invitrogen, California, USA) following the manufacturers’ protocol. We cloned the PCR products from sexual (5 individuals) and fissiparous individuals, within the latter group we included both specimens who showed double peaks in the sequences obtained directly from the PCR product (11) and some that did not show this pattern (5). Approximately ten colonies from each individual were amplified and sequenced using the T3 and T7 primers (included in the kit). Alignment was performed by sight based on the amino acid sequence, and the sequences were submitted to GenBank [GenBank: KC577271 to KC577351]. We tested whether the number of substitutions observed in the cloned sequences agreed with the expected random accumulation of mutations promoted by the polymerase errors to determine whether the variation observed in the sequences was caused by errors in the PCR process. Therefore, we assumed that errors in the PCR process followed a Poisson distribution, and the expected mean was computed using the polymerase error rate (6.3 × 10^-4^) obtained by Savolainen et al. [28] and following their procedure. We rejected the random accumulation of substitutions due to the occurrence of polymerase errors when the observed number of mutations fell outside the 99% confidence interval computed for the Poisson distribution (calculated with the DnaSP v5.10 program [29]).

In a functional protein coding sequence, more changes are expected in the 1st and 3rd codon positions. To test the functionality of the sequences obtained in this study we calculated the expected number of changes per codon site in the case of a non-functional sequence (1:1:1) and tested whether the sequences obtained from the cloned PCR products fit the expectation, using a Chi-square test.

### Nucleotide and haplotype diversity analyses

The DnaSP v5.10 program [29] was used to determine the haplotype and nucleotide diversity for each population and the species using only individuals that were not polymorphic, since we could not know the exact sequence (phase) of haplotypes for all the individuals presenting multiple polymorphic sites. Those populations for which only one sequence was available were not included. Haplotype and nucleotide diversity were also calculated for each of the cloned individuals. Two haplotype networks were constructed to study the relationships among haplotypes using TCS v1.21 [30]: (1) a haplotype network including only sequences from non-polymorphic individuals obtained after direct sequencing the PCR products and (2) a network containing only the cloned sequences from the polymorphic specimens.

### Distribution modelling

To study the possible expansion of *D. sicula* species, their potential distribution was modelled with MAXENT software v3.3.3 k [31-33] using the default settings, a random test of 25%, the threshold rule of 10 percentile training presence, and 100 replicates. The independence of 19 climatic variables were tested based on the R^2^ statistic: annual mean temperature, mean diurnal range, isothermality, temperature seasonality, maximum temperature of warmest month, minimum temperature of coldest month, temperature annual range, mean temperature of wettest quarter, mean temperature of driest quarter, mean temperature of warmest quarter, mean temperature of coldest quarter, annual precipitation, precipitation of wettest month, precipitation of driest month, precipitation seasonality, precipitation of wettest quarter, precipitation of driest quarter, precipitation of warmest quarter and precipitation of coldest quarter. Finally four independent climatic variables were used: isothermality, mean temperature of the wettest quarter, mean temperature of the driest quarter and precipitation seasonality. From 61 known localities (58 analysed in this study and 3 selected from the literature [7] –Algeria– and personal communications of L. Leria –Mallorca– and E. Solà –North of Italy–), 48 observation points were used, and 13 locations were discarded because of their geographic proximity (populations situated within the same cell of the climate layer: s13, s15, s17, s19, s20, s22, s25, s30, s32, s34, s35, s45 and s49).

## Results

### Population analyses of non-polymorphic individuals

In total, for the 58 locations sampled (Table 1), 619 sequences were obtained, and 163 (Additional file 2: Table S1) of these sequences showed double peaks in the chromatogram and were discarded for the population analyses. Because some populations had only one sequenced specimen (s04, s18, and s30) or all of their individuals exhibited polymorphic positions, only 453 individuals from 51 populations could be analysed. Only 16 populations showed more than one haplotype, and the majority of these showed two haplotypes.

**Figure 2:**
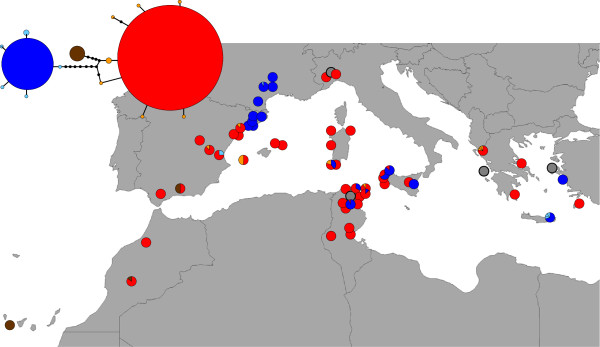
**Haplotype network and distribution in the studied populations.** Red corresponds to haplotype A, dark blue corresponds to haplotype B, and brown corresponds to haplotype C. Only haplotypes from non-polymorphic individuals are represented. The grey dots on the map show populations in which all of the specimens showed polymorphic sites.

The haplotype network was based on an alignment of 456 sequences and a length of 624 base pairs. The total number of haplotypes for the COI gene in the populations was 15 (Figure 2, Additional file 2: Table S1). The most frequent haplotype (haplotype A, the red circle in Figure 2) was observed in 292 individuals from 41 populations throughout the distribution, and presented 11 differences regarding haplotype B (the second most frequent, blue circle in Figure 2). Haplotype B was present in 144 individuals from 18 populations, the majority of which were found northwest of the Mediterranean. Haplotype C (brown in Figure 2) was only observed in six individuals from southwestern populations, and presented six differences regarding haplotype A. The other 12 haplotypes were unique or observed in only two individuals. Haplotypes A and B were observed simultaneously in 6 populations from the centre of the Mediterranean.

### Intra-individual polymorphism

**Figure 3:**
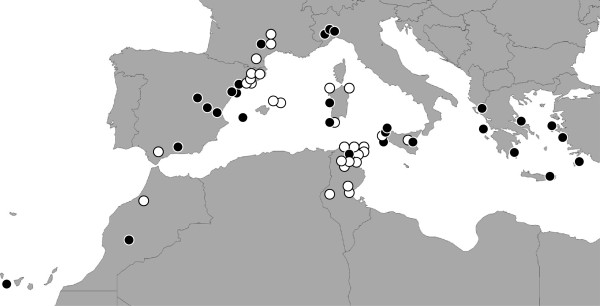
**Locations with polymorphic individuals.** The black dots represent locations with polymorphic individuals. The white dots represent populations in which all of the individuals are non-polymorphic.

Between one and ten polymorphic positions (double peaks in the sequences) were observed in 163 individuals from 29 populations (one-half of the populations, the black circles in Figure 3) when sequencing the PCR product directly. Twenty-one PCR products (11 from individuals exhibiting sequences with double peaks and 10 without) from 13 populations were cloned, and approximately 10 clones per individual (Table 4 and Additional file 2: Table S2) were sequenced to determine the haplotypes of those individuals. The final alignment had 200 sequences and a length of 537 bp.

**Table 4:** **Polymerase mistake *versus *** **heteroplasmy test**

**Specimen**	**Direct sequence (heteroplasmic positions)**	**N cloned sequences**	**N bp**	**Expected point mutations**	**Observed point mutations**	**Poisson P[X < obs]**
**s23.02**	Haplotype A	8	4296	2.7	1	0.07
**s23.09**	Haplotype A	7	3759	2.3	1	0.10
**s27.14**	Haplotype B	11	5907	3.7	14	1.00*
**s27.19**	Haplotype B	11	5907	3.7	11	1.00*
**s36.12**	Haplotype B	10	5370	3.4	7	0.95
**s36.03**^**1**^	Haplotype A	10	5188	3.0	4	0.64
**s39.08**	Haplotype A	7	3759	2.3	6	0.97
**s39.11**	Haplotype A	10	5370	3.4	7	0.95
**s39.14**	Haplotype B	10	5370	3.4	4	0.57
**s42.10**	Haplotype B	9	4833	3.0	34	1.00*
**s06.07**	Heteroplasmic (2)	8	4296	2.7	33	1.00*
**s26.12**	Heteroplasmic (1)	10	5370	3.4	31	1.00*
**s31.18**	Heteroplasmic (10)	29	15530	9.7	219	1.00*
**s31.20**	Heteroplasmic (6)	9	4833	3.0	63	1.00*
**s33.10**	Heteroplasmic (6)	4	2148	1.3	5	0.99*
**s47.03**^**1**^	Heteroplasmic (3)	9	4692	2.7	71	1.00*
**s47.11**^**1**^	Heteroplasmic (5)	10	5088	2.7	32	1.00*
**s48.04**	Heteroplasmic (4)	12	6444	4.0	95	1.00*
**s48.10**	Heteroplasmic (2)	10	5370	3.4	4	0.57
**s55.10**	Heteroplasmic (4)	3	1611	1.0	8	1.00*
**s56.15**	Heteroplasmic (7)	7	3759	2.3	26	1.00*

Expected number of point mutations for each individual based on a polymerase error rate of 6.3 × 10^-4^[28], point mutations found in cloned sequences, and probability that observed point mutations exceed the expected based on a Poisson distribution.

Sequence length was 537 bp, except in some cases marked with ^1^; N bp, total length amplified (length amplified x number of clones); *Significant differences based on 99% criterion.

In all cases we found multiple haplotypes within each individual even for those not showing polymorphic sites in the direct PCR sequence. However, some of the point mutations detected in the cloned sequences could have been an artefact of the PCR and/or cloning processes. Savolainen et al. [28] estimated this error rate to be 6.3 × 10^-4^. We compared the number of expected point mutations based on this rate with the number observed in each cloned specimen, taking into account the entire length of the amplified and sequenced DNA (the number of clones x the number of positions; Table 4). All of the individuals with polymorphic sites exhibited significant differences between the two values (except for the s48.10), which indicated that the detected point mutations could not be accounted for by the addition of erroneous nucleotides by the polymerases during the PCR reactions. None of the sexual individuals analysed had a significant result. Moreover, the cloning results showed that haplotypes A, B and C were present simultaneously in some of the specimens (Additional file 2: Table S2). However, three of the specimens in which we had not detected polymorphic sites when sequencing directly from PCR, also showed significant differences between the expected and observed point mutations (Table 4). This result suggests that, in these individuals, despite that some point mutations could be caused by polymerase error, there are most likely low-frequency haplotypes that we cannot detect in the direct sequences using PCR. In the subsequent analyses, we considered all of the individuals that showed a significant result to be heteroplasmic and the remaining individuals to be non-heteroplasmic.

**Figure 4:**
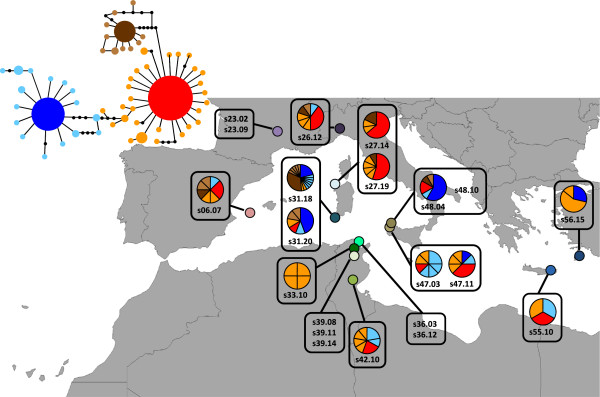
**Cloned specimen haplotype network and distribution.** The pie charts represent the haplotypes observed in each cloned heteroplasmic individual (Additional file 2: Table S2). The cloned individuals that were not heteroplasmic do not have pie charts. Red corresponds to haplotype A, dark blue corresponds to haplotype B, and brown corresponds to haplotype C. Colours of populations are as in Figure 1.

For the haplotype network reconstruction (Figure 4 and Additional file 1: Figure S2), the single haplotypes sequenced from the non-heteroplasmic individuals were discarded since those must most probably be the result of a polymerase error as shown in the previous tests. The final network contained 129 sequences and 57 different haplotypes were observed; haplotypes A, B and C were the most frequent and appeared 46, 43 and 16 times, respectively. The remaining haplotypes were unique in the majority of the cases and showed a single difference from the A, B or C haplotypes. All of the analysed individuals, except specimen s33.10 (Figure 4 and Additional file 2: Table S2), exhibited at least one of the most frequent haplotypes (A or B), and all of the individuals showed at least one unique haplotype (Additional file 1: Figure S2). The haplotype and nucleotide diversity, number of polymorphic sites and number of haplotypes were higher in heteroplasmic than non-heteroplasmic populations (*H*_*D*_ = 0.847 vs. 0.624, *π* = 0.0096 vs. 0.0017, *S* = 14.1 vs. 3.9 and *h* = 6.2 vs. 4.5, Additional file 2: Table S2).

The values of *π* for heteroplasmic individuals (0.0096) is slightly higher than that for all populations considering only non-polymorphic individuals (0.0081), and in general intra-individual *π* values are as high or even more than the *π* value found for the whole species. For *H*_*D,*_ there is an increase when we only consider the heteroplasmic individuals due to the presence of multiple haplotypes at similar frequencies.

### Predicted distribution

**Figure 5:**
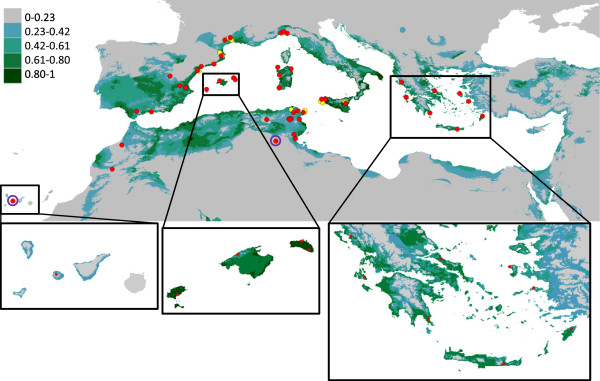
**Potential distribution of *D. sicula *in the Mediterranean Basin.** The green colour scale shows the predicted probability that conditions are suitable for the species in the studied area under the 10 percentile training presence criterion. The red dots show the observation points used for the prediction analysis, and the yellow dots show known localities discarded from the analysis (s13, s15, s17, s19, s20, s22, s25, s30, s32, s34, s35, s45 and s49). Localities surrounded by purple circle were found outside of the predicted area.

The MAXENT results show the potential distribution of *D. sicula* from the Mediterranean coasts, extending throughout the western region: northern Tunisia, Algeria and Morocco, southern Iberian Peninsula, the Italic Peninsula, and islands of the Balearic archipelago, Sicily and Sardinia. All of the populations were observed within the predicted area, except for population s44 (south of Tunisia) and s01 (Canary Islands), (Figure 5).

## Discussion

### Polymorphic sequences within individuals

Heteroplasmy, the presence of different copies of the mitochondrial genome within the cells of an individual, has been reported for several taxa [24,34-39] and was an expected outcome in the case of fissiparous flatworms, where somatic heteroplasmy would be inherited through clonal reproduction (Additional file 1: Figure S1). However, there are multiple hypotheses other than heteroplasmy that can explain polymorphic sequences; these explanations include the presence of numts or errors introduced by polymerases during the PCR reaction. In the present case, the statistical analysis (Table 4) indicated that the number of mutations observed in some individuals cannot be explained by errors introduced by the polymerases during PCR. Moreover, although the full evidence for a mitochondrial sequence to be functional and not present in the nucleus (numts) can be reached only by sequencing mRNAs (cDNAs) from the individuals [36], in our case a variety of evidence makes us think that the diverse sequences found within single planarians are not numts. Numts typically have short sequences (<200 bp) and their mutation percentages are between 5 and 25% [40-43]. The length of the shortest amplified fragments in this study was 228 bp, and the polymorphic positions observed in these fragments were also present when we amplified the larger fragment (711 bp) (Table 3). The percentage of variation for our polymorphic sequences was below 2.5%. Most of the substitutions were synonymous, and no stop codons were observed. Moreover, it is expected that the distribution of substitutions for a functional sequence shows a bias with more differences observed at the third and first position than at the second. We tested the distribution of changes in the cloned sequences of both those individuals in which the previous test could not reject that the substitutions seen are due to Taq error (non-heteroplasmic) and in those where the enzyme cannot explain the number of changes observed (heteroplasmic). The result shows that in the latter there is a significant deviation from the 1:1:1 expected ratio (Additional file 2: Table S3). However, some cases of recent numts have been described [36] that still presented the characteristics of a functional gene (were not short or did not present high levels of variation). There is still one more thing in our case that provides support for all the copies being functional, in the cloned individuals presenting heteroplasmy, we found copies of at least two or the three most frequent haplotypes (A, B and C) that are also found alone in some individuals and in whole populations, and that do not present stop codons, no traces of being non-functional (Additional file 2: Table S2).

Although fission could easily explain the coexistence of low-frequency haplotypes that differ in one or a few substitutions in a given specimen (Additional file 1: Figure S1), the origin of heteroplasmic individuals that simultaneously present haplotypes A, B and C is difficult to envision. They can be a consequence of past sexual reproduction episodes, or a result of the appearance of the third haplotype in individuals that already presented the other two. In any case, the finding of more unique haplotypes (Figure 4 vs Figure 2), differing mostly by one nucleotide from haplotypes A, B or C, in the heteroplasmic individuals supports the prediction that asexuals will tend to accumulate rare variants in all loci, which will be more accentuated in older asexuals ([44] and references there in).

On the other hand, as expected, heteroplasmy is not observed in sexual individuals. Although sexual individuals can also reproduce by fission, gametogenesis and zygote formation could represent a limitation for heteroplasmy propagation, which is a situation also observed in plants that reproduce both sexually and asexually [23,35]. However, some specimens from the s55 population from Crete possessed a copulatory apparatus and were consequently considered sexual individuals. The observation that 3 out of the 13 sequenced individuals from this population were heteroplasmic could indicate that the individuals with a copulatory apparatus were actually ex-fissiparous or that the population is composed of a mix of sexual and fissiparous individuals.

### Origin and expansion

A recent study [8] emphasised that *D. sicula* distribution could be a consequence of a relatively recent dispersion driven by human activities in accordance with a previous report [45]. In the present study we found that in *D. sicula* populations, only two haplotypes showed high frequencies, differing at 11 sites over 624 bp, and most of the populations had a single haplotype (Figure 2 and Additional file 2: Table S1). Differing from what was found in *S. mediterranea*[14], a non-overlapping geographical distribution for the different haplotypes, the most frequent *D. sicula* haplotypes are found practically throughout the distribution of the species. This lack of structure seems to reinforce the idea of a recent expansion. Moreover, the haplotype and nucleotide diversity values are extremely low at the intra-population level and when the COI values for the entire set of populations were compared to those observed in other planarian species (Table 5 and Additional file 2: Table S1), such as the freshwater *Schmidtea mediterranea*[14] or the terrestrial *Cephaloflexa bergi* and *Imbira marcusi*[27]. However, the presence of numerous heteroplasmic individuals bearing rare haplotypes in many fissiparous populations found in the present study (the intraindividual variability can be as high as the total variability of the species) suggests the ancient age of these lineages and likely for the populations where we find multiple heteroplasmic specimens.

**Table 5:** Comparison of diversity parameters between different triclad species

**Species**	***H***_***D***_	***π***	**References**
*Dugesia sicula*	0.491	0.0081	This study
*Schmidtea mediterranea*	0.773	0.0223	Lázaro *et al.*, [14]
*Cephaloflexa bergi*	0.950	0.0573	Álvarez-Presas *et al.*[27]
*Imbira marcusi*^*a*^	0.857	0.0463	Álvarez-Presas *et al.*[27]

^a^ this species has recently been renamed (Carbayo *et al.*[46]), in Álvarez-Presas *et al.*[27] appears as *Geoplana goetschi* Marcus, 1951.

Similar to other long-lived asexual lineages, *D. sicula* follows the expected accumulation of neutral or slightly deleterious substitutions over time and independent of one another, which results in high heterozygosity in their populations [47,48]. However, in the case of *D. sicula* this accumulation is found within individuals, which does not allow us to exactly quantify the levels of intrapopulation variability (unless we cloned and sequenced many individuals from each population). However, taking into account our predictions of the expected levels of heteroplasmy and of heteroplasmic individuals in populations as related to their age, we can draw some conclusions on the origin and distribution of the species.

The high frequency and allocation of haplotypes A and B appears to indicate their antiquity in the species, particularly haplotype A, which is observed from Morocco to Greece (Figure 2). In the northwestern populations (northern Catalonia and France), all 97 sequenced individuals showed haplotype B (one individual was polymorphic but likely non-heteroplasmic). The low genetic variability in this region could indicate a relatively recent colonisation of this area by individuals deriving from geographically distant populations situated in the central or eastern regions (no close population shows haplotype B, Figure 2).

Haplotype C was observed only in western populations and is most likely derived from haplotype A based on the haplotype network (Figures 2 and 4, and Additional file 1: Figure S2). Haplotype C is observed in non-heteroplasmic individuals in Morocco and the southern Iberian Peninsula and may have spread from there to the Canary Islands, where C was the only haplotype observed. Haplotype C was not present in any sexual specimen, but it was observed in heteroplasmic individuals from the western Mediterranean islands and northern Italy (Figure 4). Based on these data, we cannot determine with certainty where the haplotype originated from, but we can assume it had a relatively more recent origin than the other two haplotypes (A and B) and dispersed only through the western region. However, we can speculate that the presence of haplotype C in heteroplasmic individuals in the central regions could indicate its origin is there, and its prevalence in the s01, s02 and s05 populations could be the result of a founder effect during colonisation.

Insufficient information provided by the few most common haplotype distributions and the fact that the presence of multiple single haplotypes in a population not necessarily are a consequence of an old age for the population at that locality, make it difficult to determine where colonisation began in the Mediterranean. However, some facts can help: (1) the evolutionary sister group of this species, *D. aethiopica*[8] is distributed exclusively in Africa [49], and their split is dated around 4 My (c. 7–2 My) [20], well after the Mediterranean basin was formed and all islands had reached their present location [50,51], (2) most of the populations (either sexual or fissiparous) in which haplotypes A and B cohabit are in the central region of the Mediterranean basin (Figure 2), (3) known sexual populations originate only in either Sicily (now lost) or Tunisia and Algeria. Collectively, these data could indicate that the central Mediterranean is the region from where dispersal took place. However, the question as to how this species attained such a wide distribution with such a low dispersal capability [16,17] remains. Various factors could have contributed, including their fissiparous reproduction and their capability to resist high water temperatures.

It has been shown [52] that asexual reproduction in planarians allows a more rapid increase in population size under ecological conditions in which food is limited. Moreover, a study by Vila-Farré [53] have shown that *D. sicula* is sensitive to low temperatures and to abrupt temperature changes, while Charni et al. [10] found that the diverse Tunisian populations lived in water with temperatures ranging from 13 to 22°C. It has also been observed that during spring and autumn, higher rates of fissioning occur [54]. Therefore, they can resist high temperatures of water, and can reproduce and increase their populations very fast.

But could the species by itself reach its present distribution area? Given the maximum age of the species situated around 4 My (c. 7–2 My) [20], when the Mediterranean basin was already formed and its islands in its present situation, it seems difficult to explain how the animals could have moved between islands and the continent (Corsica, Sicily, Tunisia…) even in one direction or the other. If the origin of the species preceded the Messinian Salinity Crisis or else as a consequence of the sea level changes during the Pleistocene glaciations, we could maybe hypothesize the contact among some fluvial basins in the central Mediterranean that would allow its expansion there, but still this would not explain its presence in places situated as far away as the Greek islands, the Canary islands or how haplotype B could have reached the French coast. These latter cases provide support for a mixed history of self-dispersal and human introductions. De Vries [55] already proposed that given the antiquity of human trade in the Mediterranean basin there may have been multiple opportunities for these animals to be transported around, including modern commercial trade of fish, plants or timber, as has been proposed for other planarian species [56-58], which also could explain this expansion.

Our hypothesis is then that *D. sicula* most likely originated in northern Africa and dispersed through the Central Mediterranean region. After one or multiple populations became triploid and fissiparous, they colonized the whole Mediterranean basin either by their own means or assisted by human activities (probably both scenarios occurred).

### Species distribution and future expansion

Under the hypothesis that *D. sicula* is a relatively recent coloniser, it was advisable to test whether this species had reached its current range of fitted distribution or was still expanding. The potential distribution predicted with the climatic models for *D. sicula* (Figure 5), fits well with what has been observed, mainly occupying coastal regions. It has been reported that *D. sicula* does not cope well with abrupt changes in temperature [53] and is typically observed near the coast, where the water temperature is highest in the winter and the thermal changes are less abrupt than in the high mountains. All known populations, except for s44 and s01, have been observed within the predicted area; however, population s44 inhabited an oasis in the Tunisian desert, the special conditions in this reduced area could be ideal for the species although precision of climate layers used in the analysis do not allow for detecting them. Population s01 in the Canary islands is found within Garajonay National Park, in a area of laurel forest, thought to be the remnant of subtropical woods that covered the Mediterranean area during the Tertiary period, which likely provides *D. sicula* the conditions needed to survive, although the models, again as a consequence of the scale, may have not been able to detect it.

Some regions that are potentially suitable for *D. sicula* have not yet been widely sampled, such as northern Africa (except for Tunisia and some regions of Algeria and Morocco), the southwestern Iberian Peninsula and parts of the Italic Peninsula. However, restriction pattern analyses showed similarities among some populations north of the Italic Peninsula and *D. sicula*[59,60], although the Italian populations could not be identified; moreover, Benazzi and Deri [61] reported populations in Tuscany (including the island of Pianos), Ponza Island (between Rome and Naples), Rome (Lazio) and Calabria that could be attributed to *D. sicula* based on morphological features, and some populations with a chromosomal complement of 27+ 2B chromosomes have been reported in Calabria by Deri et al. [62]. In other areas, *D. sicula* has never been reported (Corsica) or is relegated to the periphery (Sardinia), although these regions appear to fulfil the climatic requirements of the species. However, several populations of *D. benazzii* have been observed in these regions. The competition between *D. sicula* and *D. benazzii* may explain why *D. sicula* is absent in Corsica and has a low presence in Sardinia. However, *D. sicula* is present together with other planarian species in localities such as Lebna in Tunisia (*S. mediterranea*), which points to the possibility that historical reasons could also explain its absence in some regions (they may have never been introduced by humans or had the opportunity to reach by their own means).

For the eastern Mediterranean, some populations described in Israel as Type 4 by Bromley [63] showed karyotypes and morphological characteristics similar to those of *D. sicula*. Although Type 4 was related to *D. biblica* in a study by Bromley [64], these two species cannot be definitively differentiated [12]. An accurate study of these two species is necessary to clarify their status and confirm whether *D. sicula* is also present in that region or whether the presence of a closely related species potentially precluded its colonisation.

Recent exhaustive samplings performed in the Iberian Peninsula in search of *Dugesia subtentaculata* (M. Vila-Farré and L. Leria personal communication and one of the authors, MR), allows us to confidently state that *D. sicula* is only observed on the east and south coast, as the prediction analysis suggests. In this area, *D. sicula* cohabits with other freshwater planarian species such as *S. mediterranea*. In recent years, populations of *S. mediterranea*[14] appeared to have vanished, whereas populations of *D. sicula* became more frequent. Additionally, in the Greek population from Rhodes (s56), *D. sicula* cohabits with the autochthonous *D. elegans*, and only a few animals belong to this latter species. This result could indicate that *D. sicula* can have an adverse effect on the native triclad species, which contrasts with what appears to occur in Sardinia. A similar situation has been reported in Wales, where the presence of two immigrant species, *Planaria torva* and *Girardia tigrina* has been followed via three surveys over 50 years [56,58,65,66]. The most recent report [57] indicates that the invasion of *G. tigrina* has been more successful, which is most likely because of its asexual reproduction and opportunistic feeding characteristics, although other factors, such as its capability to adhere to surfaces better than other triclads, have also been considered. Moreover, *G. tigrina* has been shown to have a negative effect on native species of triclads, which is most likely explained by food competition.

## Conclusion

In conclusion, it appears that *D. sicula* has reached a large proportion of the area of its potentially favourable distribution in the Mediterranean basin, being a remarkable case of a broad colonisation, in extreme contrast with the rest of Mediterranean *Dugesia* species, with all of them being endemic or with very restricted distributions. *D. sicula* expansion is now limited to spreading to new freshwater basins within the areas it currently inhabits. However, future changes increasing the temperature, such as those predicted by climate change hypothesis, could expand its fitted area to more northern and interior areas.

## Competing interests

The authors declare that they have no competing interests.

## Authors’ contributions

MR and EML designed the study. EML performed the molecular work and obtained the sequence data. EML and MR conducted all of the analyses and wrote the manuscript. Both authors read and approved the final manuscript.

## Supplementary Material

Additional file 1: Figure S1 Schematic representation of the spreading of a new mutation in fissiparous organisms. The yellow and orange colours represent the tissues bearing the new mutations. The arrows represent fission (black) and regeneration (blue) cycles. **Figure S2.** The haplotype network based on cloned sequences. Each circle represents a haplotype observed in the sample. The black dots represent intermediate (non-present) haplotypes, the lines connecting the haplotypes (either present or not) represent one nucleotide change, and the size of each circle is proportional to the haplotype frequency in the sample. The colours in the network correspond to those on the map and represent the studied locations. The crossed-out names of the individuals represent non-heteroplasmic specimens, and their sequences were not included in the network. Click here for file

Additional file 2: Table S1 Genetic diversity in the populations. **Table S2.** Genetic diversity in the cloned individuals. **Table S3.** Chi-square test for the distribution of substitutions along the three codon positions (null hypothesis equifrequent distribution, 1:1:1). Click here for file
