# Supplementary material for: Dugesia sicula (Platyhelminthes, Tricladida): the colonizing success of an asexual Planarian
Source: BMC Evol Biol. 2013 Dec 11;13:268. doi: 10.1186/1471-2148-13-268 (PMC3922848; doi:10.1186/1471-2148-13-268)
Supplement: Additional file 1: Figure S1 — Schematic representation of the spreading of a new mutation in fissiparous organisms. The yellow and orange colours represent the tissues bearing the new mutations. The arrows represent fission (black) and regeneration (blue) cycles. Figure S2. The haplotype network based on cloned sequences. Each circle represents a haplotype observed in the sample. The black dots represent intermediate (non-present) haplotypes, the lines connecting the haplotypes (either present or not) represent one nucleotide change, and the size of each circle is proportional to the haplotype frequency in the sample. The colours in the network correspond to those on the map and represent the studied locations. The crossed-out names of the individuals represent non-heteroplasmic specimens, and their sequences were not included in the network. [file 1471-2148-13-268-S1.pdf]

Additional file 1: Figure S1

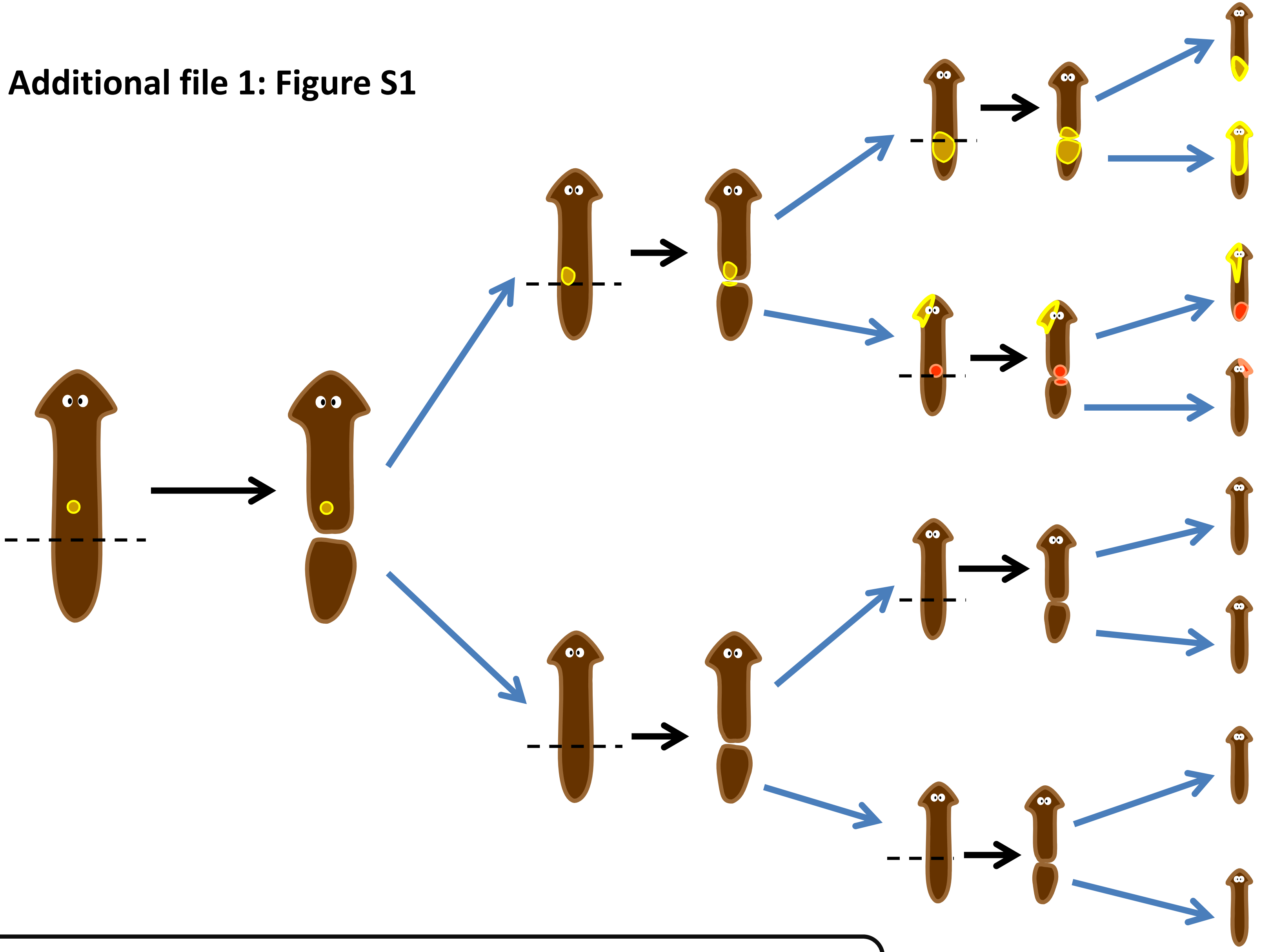

Schematic representation of the spreading of a new mutation in fissiparous organisms. The yellow and orange colours represent the tissues bearing the new mutations. The arrows represent fission (black) and regeneration (blue) cycles.

Additional file 1: Figure S2

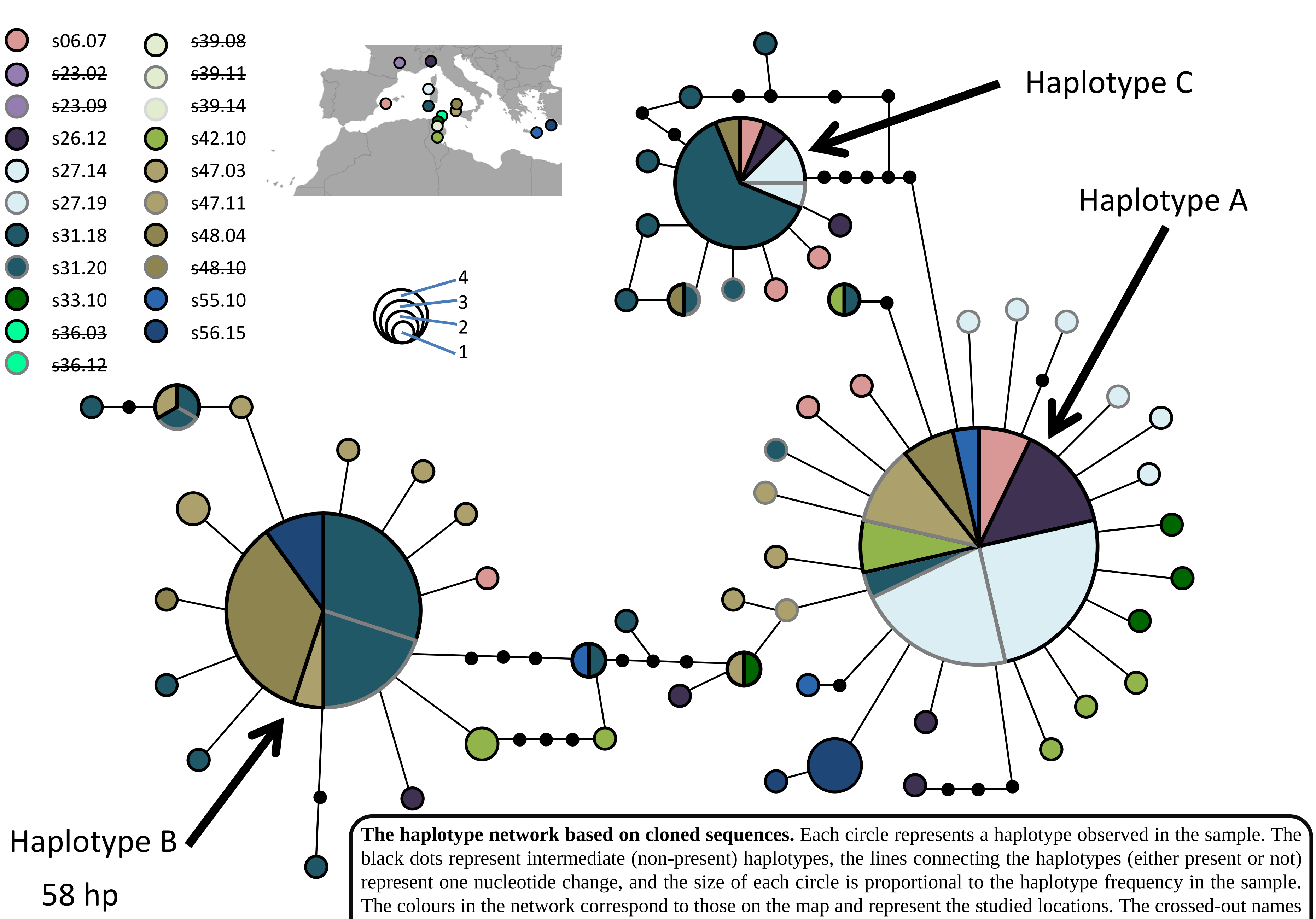

The haplotype network based on cloned sequences. Each circle represents a haplotype observed in the sample. The black dots represent intermediate (non-present) haplotypes, the lines connecting the haplotypes (either present or not) represent one nucleotide change, and the size of each circle is proportional to the haplotype frequency in the sample. The colours in the network correspond to those on the map and represent the studied locations. The crossed-out names of the individuals represent homozygous specimens, and their sequences have not been included in the network.
